# Scaling up the impact of melanoma advocacy: an interview with Bettina Ryll

**DOI:** 10.1242/dmm.052202

**Published:** 2024-12-24

**Authors:** Bettina Ryll

**Affiliations:** Melanoma Patient Network Europe


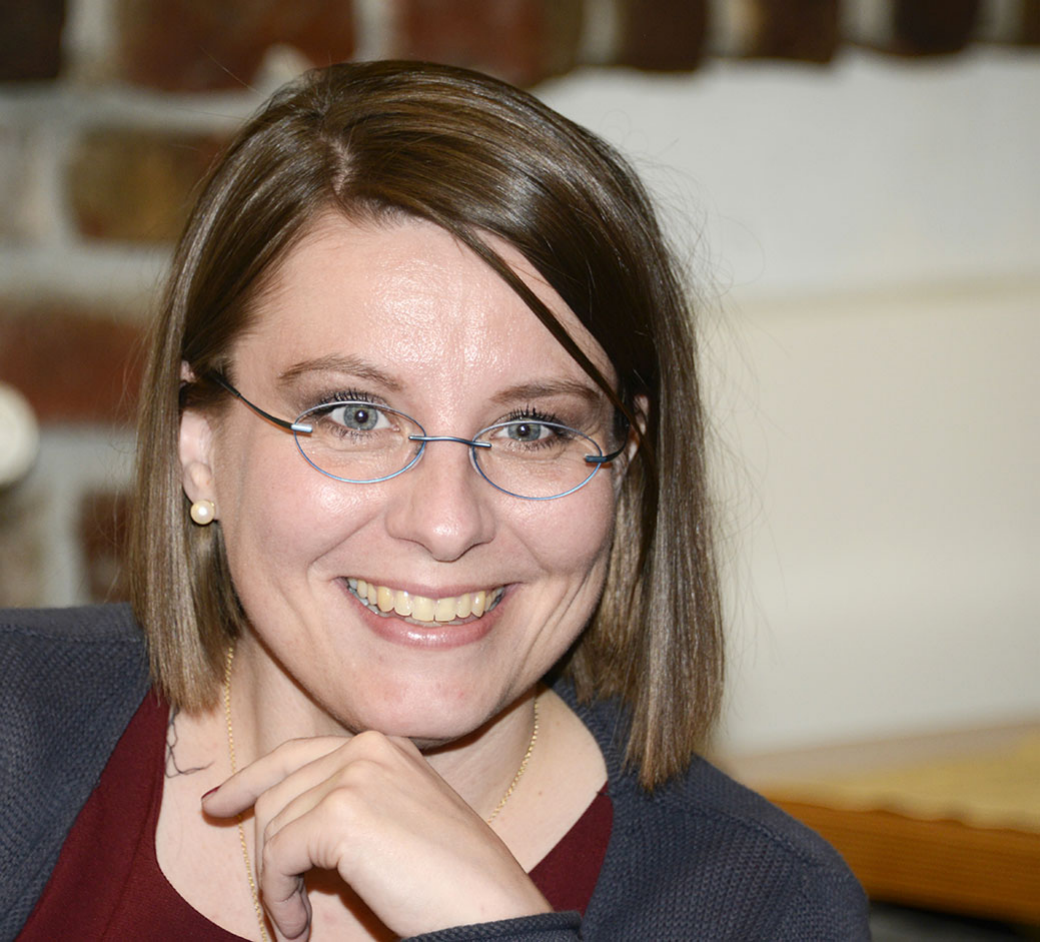


**Bettina Ryll**

Melanoma is a type of cancer that originates from the melanin-producing cells of the body, called melanocytes. Since 2011, 11 new therapies that are greatly improving survival rates have been approved for advanced melanoma. Despite these promising advances, the number of new invasive melanoma cases diagnosed annually increased by 27% during the past decade (2013–2023). In 2024, more than 100,000 people are expected to have been diagnosed with melanoma in the USA alone – with ∼8000 deaths, indicating that more needs to be done to help patients with this cancer.

Bettina Ryll is a research scientist and physician by training who has dedicated more than a decade to patient advocacy as the founder of the Melanoma Patient Network Europe (MPNE; Box 1). She was the first non-oncologist to chair the European Society for Molecular Oncology's (ESMO) Patient Advocates Working Group and a member of the first EU Cancer Mission Board. She works as a strategist at the Stockholm School of Economics Institute for Research (SIR), where she focuses on precision medicine implementation. In this vein, she also acts as a strategist for Vision Zero Cancer to drive progress in personalized medicine and has been involved in numerous European initiatives on precision medicine implementation. Here, she discusses her personal experience and motivation in patient advocacy, and her strategies to expand the impact of her work to benefit all people with melanoma.

**Box 1. Melanoma Patient Network Europe**

Melanoma Patient Network Europe (MPNE) is a not-for-profit organisation that supports patients with melanoma and their families and, ultimately, aims to solve melanoma. The network was officially launched in 2014, following an earlier project, the Melanoma Independent Community Advisory Board. Its core goal is to promote and improve prevention, early detection and effective therapies for melanoma, and to enhance patient care for everyone with the disease. The MPNE provides crucial education and resources for patients and their families to inform and empower them in their medical journey. Furthermore, it has developed a systematic and evidence-based approach to patient advocacy work, which has led to tangible improvements in fundamental and clinical research for melanoma. From organising events to facilitate collaboration between stakeholders to being actively involved in European research policy and funding decisions, MPNE is driving change for melanoma research and care.

## Could you tell us about your career – having trained as both a clinician and basic researcher – and how this benefits your advocacy work?

I don't think that any one of us grows up with the dream of becoming a patient advocate. I definitely didn't – I wanted to be a paediatric surgeon. You end up in patient advocacy because of a personal connection, usually because something happened to you or someone close to you. In my case, it was my husband, who died of melanoma in 2012.

In terms of my background, I had studied medicine in Germany and France, and then moved to University College London (UCL), UK for a basic research PhD on a topic that was really fascinating. There I met my late husband, and both our daughters were actually born in the UK. I then got offered a postdoc position in Sweden, where I still live now. I really enjoyed my time in basic research, although I had kind of hoped to be working more at the interface of research and medicine – that was actually one of the reasons for moving to Sweden, as it was the time when SciLifeLab was established as an interface between molecular biology and medicine. However, everything changed the moment my husband was diagnosed with metastatic melanoma out of nowhere in 2011.

I thought I understood what medicine was about and had this, maybe naïve, assumption that medicine was doing the best for its patients and for research. As a PhD student at UCL, I went through rigorous training, and was taught how to be critical and how to analyse science. But then when we were relying on experimental medicine and, in particular, clinical trials for my husband – because it was before the new drugs for melanoma were approved – I started looking at science differently. We were on the receiving end of the healthcare system and clinical research, and I realized that a lot of what I thought to be great science, actually, wasn't that great at all once you are on the receiving end of it.

My background gave me a very different perspective. I think I had a very high moral expectation of the healthcare system that I didn't see fulfilled. At the same time, I had technical expertise, and I had the professional knowledge. I never really saw myself as patient advocate. I didn't want to have a patient organization either. I guess it just happened gradually – it began because we were on patient forums to look for information for my husband, and I realized that people didn't understand the science behind the disease because either they couldn't access it, or they read it and didn't understand it. So, I started explaining the science and could see how much difference it made to them. Now, looking back, I've always been teaching throughout my career, so my background has, definitely, influenced how I look at advocacy. I didn't feel bound to what other people think patient advocacy should be because we were so close to it, I could see where the gaps were, where the errors were, where things had to change – and I just focused on that.

When I was chairing the European Society for Medical Oncology (ESMO) patient advocacy working group between 2015 and 2018, we were actually asked “What is a patient advocate?”. You wouldn't believe, but there was no real definition for it at the time. Initially, there was a lot of disagreement but, in the end, I proposed something simple that hasn't been challenged so far. So, the first point is personal experience − such as being a patient with personal disease experience or being the carer of a patient. This is someone who understands the disease by living it, which is important. However, there is a difference between a patient and a patient advocate because an advocate cares about the group of people with their condition and they can reflect on the experiences of the group, not just their own experiences. Often, they can tell you a lot about how communities work, what people are worried about, what impacts their lives and what the issues are. At this point, you are already a patient advocate. But the third dimension is having expertise that is linked to your professional background. For me, this was medicine and research; but we also have lawyers, who look at the interface between patients and, for example, patient rights. Or we have artists, who look into very different ways of dealing with a patient experience. So, there is an intersection between professional background and personal experience of a disease. There is no one-size-fits-all solution in patient advocacy. It's more about getting going and seeing how you can make things better.

## How did you to start setting up Melanoma Patient Network Europe?

When I started, there was no European melanoma patient organisation. There were local groups looking into prevention and early detection because that was the only thing we could do back then. When I say ‘back then’, it was only like 2011, which is not that long ago. Then when the new drugs were coming, we decided that we had to get organized and set up the Melanoma Patient Network Europe.

Then, at some point, everyone I had been working with was dead and I was the only one left, because I wasn't a patient myself. I had a full-time job, I was just widowed and I had two small kids, so becoming an advocate wasn't the plan. I had never organized a conference, I had never done anything like this − and I, definitely, didn't want to be an advocate. But I was just not willing to let it all go to waste. Then I thought, “Okay, what's the biggest issue for us right now?” − which was the way clinical trials were designed − and that's where it all began. I started by looking at the problems, and then I thought about who could help us or who was the source of the problem, and just invited them to the conference. That was the basis for the first conference, and we already had representatives from the European Medicines Agency and the UK Health Technology Assessment (HTA) body NICE there. Things developed from there and, before we realised, we were involved in research projects and policy initiatives.

When I started in advocacy, I was very angry and I was upset, and I thought there was one person, somewhere, whose fault it was. Now, in hindsight, it seems so naïve; but I really assumed that there somehow was a single person or party responsible for the failings in the healthcare system – and, actually, many others still act like this today. Once you go looking, you realize it's not like that. You go from one stakeholder to the next, and they all have a coherent way of thinking and acting. A lot of them are also really nice people, who really care. Often, they are in this position because they themselves have lost someone to cancer or have had cancer themselves. So, it's not that easy that there's someone who is the cause of all the problems. It is more that the alignment between the pieces doesn't work, and that took me a few years to figure out.

The only way to go about that is to change the system and make the default care good, so that independent of whether you know us or are interested in the science, you get good care.

## In your advocacy work, what is your main goal?

I found it upsetting that, if you didn't speak English, if you didn't have access to the papers and you weren't scientifically or medically trained, it was very difficult for you to understand the research. But then there's only so much you can do by supporting individuals. You make their life better, but it has no scale. It still requires willingness on the other side to learn, so you can only help those who are willing and able − and that wasn't good enough. I started a Facebook group but I still wasn't reaching everyone and, ideally, we want to keep everyone safe. The only way to go about that is to change the system and make the default care good, so that independent of whether you know us or are interested in the science, you get good care.

When I first encountered patient advocacy, ∼10 years ago, the overall situation was very different. Patient engagement was a tick box at best and you were invited to ‘tell your story’. I thought this was so pathetic. So, when I started with patient advocacy, I wanted to move forward in a very focused way, to make sure what we were doing was, actually, changing something. I began by focusing on clinical trial design, and I would put feedback and evaluation of trial designs on patient forums. I still do this sometimes, but less so, because they're not as dreadful as they were and also because my primary intent was to inform patients, but then, of course, it did affect trial recruitment and retention.

So then, all of a sudden, we were involved in somewhat more intelligent and meaningful talks about trial design with the pharmaceutical industry and academic researchers. We started to have more influence because, it turned out that, those trial designs that had been adapted because we had commented on them recruited much, much better − and people loved them. Moving on from this, we started working with research funders. So, instead of talking to the researchers, we evaluate their grants, which is much more efficient. We also help to write grants and provide training to write grants. For example, we are participating in EU grants, and we're getting pretty good at it.

Now, when we interact with researchers, we are in a different type of role, and we have something to offer. We have been involved in projects from the trial-stage to drug approval, to HTA approval, then to uptake in the healthcare system. So, now we have a lot of insight into research implementation into healthcare systems. We understand what can go wrong during this process and what you can do to anticipate that. We know how you can be faster and smarter in this process, and to whom you should you talk for specific support. Because of this, people come to us, and we go into a research partnership with a very different mindset. We look at the project more proactively, thinking about how we can contribute.

When we have meetings with various stakeholders, we describe where we see the issues as patient advocates. Having these discussions has already changed a lot for melanoma research and care. Then, of course, you have another layer of complexity, because everyone's story is different, and no one story is necessarily representative of all patients. We now use problem-solving processes like design thinking to capture diversity in a group of patients. So, when we started, we were just trying to help a single patient at a time, but now we have a more systemic approach that then came with new challenges, but you learn from these challenges as a larger, multi-stakeholder community that has changed things in the end.

If you just share a little bit more, others move forward as well and everyone benefits. That's the approach we're taking, and I hope to do more of that going forward.

## How else do you expand your impact on melanoma research?

We start by looking at what affects our patients and where the need is, and then build backwards. Then − as a community, not just patients but all of us together − we discuss how we should tackle this. From our conferences, we've had research collaborations and new research ideas, by facilitating networking around a shared purpose. We then build on this, for instance, by looking into research proposals with a more targeted lens. I was on the first EU Cancer Mission Board, so I spent quite some time on EU Research Policy. Because I understand what calls for research are looking for, I can help people to find each other and facilitate collaborations.

There are also many countries in Europe with less research activity, referred to as widening countries. In these countries, there can be limited funding available to start with, but capacity to absorb funding can also be an issue. For instance, you can get research funding into a country, but they may not be able to find people to hire. This is where we need to increase research capacity, and it's important because it leads to better access to clinical trials and experimental medicine for patients in these countries. This has a direct impact on their care because clinicians involved in research start thinking differently. This is something that isn't melanoma specific.

Many issues that we face are not melanoma specific. I was in the patient advocacy working group at ESMO and then on the EU Mission Board, which were both pan-cancer. We've always been trying to bring people together and look at it from the meta-level. It just doesn't seem right to focus all that energy on your own disease. If you just share a little bit more, others move forward as well and everyone benefits. That's the approach we're taking, and I hope to do more of that going forward.

### As we discussed, there have been massive leaps in terms of treatments for melanoma recently, but what do you think are the outstanding concerns for patients and where do you hope melanoma research will progress to in the next 5 or 10 years?

I think prioritization is the absolute worst thing one can do in this type of setting, because we have what I call a ‘problem carpet’. To address this, we use tools that lean startups use because we have similarities − like our need for speed and limited resources. By using these tools, we map the patient pathway to pick up where the problems are. Depending on the stage of disease, you have very different issues. To me, the issues are all valid because it affects people, and we should be doing right by people. In the early stages of the disease, there is a huge psychological impact because of the uncertainty. You're likely to survive if you're diagnosed at that stage, but not everyone survives, and if it progresses you still have a real threat of dying. So, there's the psychological component. We also need to identify the patients who have a high risk of their disease progressing. We don't want to treat everyone because if we overtreat, we induce toxicity. So, we need to implement risk stratification to minimise intervention, both on the surgical side but also on a therapy side. I think this is an exciting place.

Then in stage three melanoma, we have seen that there is a difference between neoadjuvant and adjuvant immunotherapy treatment. In the first publication by Christian Blank, you could see that there was a difference in T-cell activation if you use immunotherapy before taking out the regional lymph node. There seems to be a spatial-temporal component that depends on when they're giving an infusion and when they do the surgery. I think that's a super interesting field to improve our understanding of how our body actually mounts an effective immune response and the sequence of these events. This is where we loop back into more basic science, so that we understand the disease more before we intervene. For 10 years, everyone has been looking for a magical biomarker to predict response to immunotherapy but it's not working. I believe that, to solve this, we have to dig deeper into the science, both in oncology and in immunology. We also have to think about what else influences response to therapy, like the microbiome and nutrition. Ultimately, we need to dig deeper into the fundamental science of pathophysiology to progress.

Then there is where my personal heart is. My husband was diagnosed at stage four, out of nowhere. Survival was below 5% for people at stage four when my husband was diagnosed and died. Now it's about 50%, but that still means that 50% of people die; and that − for me − is not acceptable. This is the hard end of the disease and it is why we fear cancer, because this is where people die. And so, we need options for stage four. We are now organizing a meeting in December in Berlin that focuses on advanced melanoma. Obviously, if we're getting better at diagnosing and treating people at the early stages of the disease, hopefully, we will have fewer people arriving at the advanced stage where there's just nothing that seems to work anymore. It's always easy to say that we should have prevented the disease or we should treat earlier, but patients still end up at stage four, and we're not intelligent enough to solve this stage.

In my other life, I have a position at the Stockholm School of Economics Institute for Research, where I work in precision medicine and implementation. Also, I've just been involved in WHO Europe's novel medicines platform. We submitted a proposal for a demonstration project on precision medicine implementation across Europe, which has just been accepted. This is something that helps not just melanoma but all cancers. The current precision medicine approach just gives options for a few patients right now but I think, in the future, we have to layer and refine additional options by expanding research. For example, we're talking to companies developing organoids for drug testing and combination therapy testing. We need to think about the most appropriate model systems for research so we can develop better therapy choice, especially for advanced stage cancer.

## Conclusions

There are various approaches to working as a patient advocate that can build on people's existing expertise. Harnessing and gathering these skills in patient organisations, such as MPNE can lead to true change in research and patient care. Melanoma is a unique disease that entails drastically disparate levels of risk depending on the stage at which the cancer is diagnosed. Here, Bettina outlines how advocacy work and future research need to benefit people facing every stage of disease to ensure their differing problems and needs are met. It is a challenge in itself to scale up advocacy work to impact everyone. However, by creating a dynamic and international network of stakeholders, and using evidence-based approaches, it is clear that substantial outcomes can be achieved.

